# Humanized anti-Sialyl-Tn antibodies for the treatment of ovarian carcinoma

**DOI:** 10.1371/journal.pone.0201314

**Published:** 2018-07-27

**Authors:** David A. Eavarone, Linah Al-Alem, Alexey Lugovskoy, Jillian M. Prendergast, Rawan I. Nazer, Jenna N. Stein, Daniel T. Dransfield, Jeff Behrens, Bo R. Rueda

**Affiliations:** 1 Siamab Therapeutics, Inc., Newton, MA, United States of America; 2 Vincent Center for Reproductive Biology, Department of Obstetrics and Gynecology, Massachusetts General Hospital, Boston, MA, United States of America; 3 Harvard Medical School, Boston, MA, United States of America; 4 Morphic Therapeutic, Waltham, MA, United States of America; University of South Alabama Mitchell Cancer Institute, UNITED STATES

## Abstract

The expression of Sialyl-Tn (STn) in tumors is associated with metastatic disease, poor prognosis, and reduced overall survival. STn is expressed on ovarian cancer biomarkers including CA-125 (MUC16) and MUC1, and elevated serum levels of STn in ovarian cancer patients correlate with lower five-year survival rates. In the current study, we humanized novel anti-STn antibodies and demonstrated the retention of nanomolar (nM) target affinity while maintaining STn antigen selectivity. STn antibodies conjugated to Monomethyl Auristatin E (MMAE-ADCs) demonstrated *in vitro* cytotoxicity specific to STn-expressing ovarian cancer cell lines and tumor growth inhibition *in vivo* with both ovarian cancer cell line- and patient-derived xenograft models. We further validated the clinical potential of these STn-ADCs through tissue cross-reactivity and cynomolgus monkey toxicity studies. No membrane staining for STn was present in any organs of human or cynomolgus monkey origin, and the toxicity profile was favorable and only revealed MMAE-class associated events with none being attributed to the targeting of STn. The up-regulation of STn in ovarian carcinoma in combination with high affinity and STn-specific selectivity of the mAbs presented herein warrant further investigation for anti-STn antibody-drug conjugates in the clinical setting.

## Introduction

Ovarian cancer is the most lethal gynecologic cancer in the United States[1] and despite surgical debulking and chemotherapy, the five-year survival rate remains below 50%. This lack of clinical success has led to the integrated genomic analysis of ovarian cancer by The Cancer Genome Research Network[2]. The result of this analysis highlighted the heterogeneity of the disease and further supported the concept that ovarian cancer has relatively few ubiquitous targetable mutations, amplifications or deletions. More recently, investigators have focused on identifying antigens present on ovarian cancer cells that could serve as targets to deliver cytotoxic payloads[3,4]. Antibody drug conjugates (ADCs) that recognize tumor cell specific antigens provide selectivity for delivery of highly toxic anti-cancer agents which would not otherwise be able to be delivered in a safe mannner [5]. By example, pre clinical and clinical studies support the concept that monomethyl auristatin E (MMAE), a potent anti-mitotic agent, could potentially be effective agent against ovarian carcinoma[6,7],[8]. While effective, MMAE is too potent to be delivered in non-targeted form. Therefore, identifying alternative ovarian cancer cell surface antigens and developing improved strategies for targeting ovarian cancer via ADCs are warranted.

Aberrant forms of glycosylation exist across a range of solid tumors including ovarian, bladder, breast, cervical, colon, and lung cancer[9–13]. Tumor-associated carbohydrate antigens (TACAs) have been demonstrated to be specific and suitable for selective tumor targeting[14–18]. The cancer-specific Sialyl Thomsen-nouveau (STn) antigen (Siaα2-6GalNAc-α1-O-Ser/Thr, also known as CD175s) is formed through activity of the sialyltransferase ST6GalNAc-I[19] upon the Thomsen-nouveau (Tn; GalNAc-α1-O-Ser/Thr) antigen. Core 1 synthase (T-synthase, encoded by *C1GALT1*) and its specific chaperone COSMC (core 1 β3-Gal-T-specific molecular chaperone, encoded by *C1GALT1C1*[20]) compete with ST6GalNAc-I for the Tn substrate for extended O-glycan carbohydrate synthesis[21]. However, an increase in ST6GalNAc-I activity and/or a loss or mutation of *C1GALT1/C1GALT1C1* results in sialylation of the core GalNAc and subsequent increase in STn expression[19]. Elevated ST6GalNAc-I levels may result in *de novo* STn expression and the induction of a more malignant behavior in carcinoma cells[22,23]. An increase in STn promotes tumor cell invasiveness and metastatic properties as well as resistance to chemotherapy[24,25]. In addition, STn enables tumors to evade the host immune system[26]. The functional properties of STn and its increased expression in ovarian cancer suggest the elimination of STn positive tumor cells may impact tumor growth and offers the potential for important clinical benefit to patients. Previous attempts to target STn in the clinic have been made utilizing a synthetic cancer vaccine, but efficacy has been limited[27,28] using this modality. Post-hoc analysis of study data showed an association between STn antibody titer and tumor response, supporting the idea that an antibody-based immunotherapy could offer clinical benefit[29].

We previously reported the identification and characterization of novel murine anti-STn antibodies[30]. These antibodies show high affinity and specificity for the glycan itself, independent of conjugated protein, and as such represent a potential therapeutic tool for human carcinomas that express STn[18]. Herein, our objective was to develop humanized variants of these anti-STn antibodies, conjugate them with MMAE and assess their efficacy with *in vitro* and *in vivo* preclinical models of ovarian cancer. We validated the further clinical development of this therapeutic through tissue cross-reactivity studies and cynomolgus monkey toxicity evaluation. We demonstrate here for the first time that humanized anti-STn-MMAE conjugates provide a uniquely glycan-specific and effective targeting mechanism for potential treatment of ovarian carcinoma.

## Materials and methods

### Antibodies and humanization

2G12-2B2 and 5G2-1B3 are murine antibodies developed previously[30]. To humanize, the sequence of each antibody was compared with human germline genes using the IMGT/V-QUEST online tool (IMGT®, the international ImMunoGeneTics information system® http://www.imgt.org (founder and director: Marie-Paule Lefranc, Montpellier, France)). The structure of each domain was modeled using BioAssemblyModeler (BAM) and visualized using Pymol to select mutation sites as previously described[31]. The humanized VH and VL genes were synthesized by DNA 2.0 (Newark, CA) and were recombinantly expressed in HEK293T cells (MIGS, Cambridge, MA). For comparison, an irrelevant (anti-respiratory syncitial virus F-protein binding) human IgG1 antibody was generated and used as an isotype control.

### Qualification of STn specificity using glycan array

A cancer discovery-focused glycan array was used to determine the glycan binding specificity of anti-STn antibodies[30]. Glycans included 25 matched Neu5Ac/Gc pairs (including 9-O acetylated versions) along with several un-sialylated versions. Epoxy slides were blocked (0.1 M Tris, 0.05 M Ethanol amine, pH 9.0) for 1 hour at 50°C. Slides were washed with distilled water, and blocked again with 1x PBS containing 1% (w/v) ovalbumin (OVA) for 1 hour. Blocking buffer was aspirated and anti-STn antibodies were added at 1 μg/mL concentration diluted in blocking buffer for 1 hour. Slides were washed twice with 1x PBS containing 0.1% (v/v) Tween, then once with 1x PBS alone. Next, secondary antibody (Cy3 goat anti-human, Jackson ImmunoResearch cat# 109-165-098) was added at a final concentration 1.5 μg/mL in 1x PBS for 1 hour. Slides were washed with 1x PBS then distilled water and air dried before reading on the GenePix 4000B (Molecular Devices). Fluorescence intensities were measured and background was subtracted using GenePix Pro software. STn-specificity was determined comparing STn vs. non-STn glycan binding. Intensity of STn (Neu5Acα6GalNAcαR and Neu5Gcα6GalNAcαR), 9-O acetylated STn (Neu5Ac9Acα6GalNAcαR and Neu5Gc9Acα6GalNAcαR) and Tn (GalNAcαR) were compared along with the remaining 66 glycans on the array. R is the linker ProNH_2_ (O(CH_2_)_2_CH_2_NH_2_).

### Quantification of STn at the cell surface by flow cytometry

The binding affinities of anti-STn antibodies to STn on the surface of tumor cells were determined by flow cytometry. Anti-STn antibodies were screened for binding to MDA-MB-231 cells stably transfected with ST6GalNAc-I to express STn and for the absence of binding to non STn-expressing parental cells. Cells were harvested using StemProAccutase buffer, resuspended to a concentration of 5x10^6^ cells/mL in staining buffer (5% (v/v) heat-inactivated fetal bovine serum (FBS) in 1x PBS). Fifty microliters of cell suspension and 50 μL of staining buffer with anti-STn antibodies were combined. Antibodies were screened using a serial dilution over a concentration range of 0–300 nM. Antibodies and cells were incubated for 1 hour at 4°C. Cells were washed three times with staining buffer and binding of antibodies was determined using an anti-human IgG allophycocyanin (APC)-conjugated secondary antibody at 1:1500 (Southern Biotech cat# 9042–11) diluted in staining buffer. A total of 5,000 events were acquired per sample using the BD Accuri C6 and data was analyzed using FlowJo software. Mean APC fluorescence and % APC positive cells were calculated. These data were log transformed then fit to a nonlinear regression model to obtain a dose response curve and EC_50_ binding calculations using GraphPad Prism software.

### ADC conjugation

A subset of humanized variants of anti-STn antibodies with promising binding attributes were conjugated to a cathepsin B-labile maleimidocaproyl-valine-citruline-p-aminobenzyloxycarbonyl-monomethyl auristatin E (MC-vc-PAB-MMAE, referred to as CL-MMAE in the text) by Moradec (San Diego, CA) or ADCBio (Denbighshire, GB) using standard methods. Antibody inter-chain disulfide bonds were reduced and the maleimide linker was attached to the reduced cysteines. Conjugated antibodies were purified with a Sephadex G50 column; drug-antibody ratio and conjugation efficiency was determined for each antibody by hydrophobic interaction chromatography (HIC).

### Cytotoxicity assay

Cells were plated between 1,000 and 8,000 cells per well and allowed to attach overnight. Conjugated anti-STn antibodies were added to wells at select concentrations (100, 80, 60, 40, 10, 5, 1 and 0.5 nM). An irrelevant isotype control and unconjugated antibodies were used as controls at the same concentrations. ADCs were incubated with cells for 72 hours to generate a killing curve. Per manufacturer’s instructions, Promega ADC CellTiter-Glo® Luminescent Cell Viability Assay kit (cat# G7570) was used to determine the amount of ATP present, an indicator of metabolically active cells. Equal volume of CellTiter-Glo® reagent was added directly to cell cultures. The addition of this reagent resulted in cell lysis and generation of a luminescent signal proportional to the number of live cells present in the culture. Luminescent signal was determined and relative ADC potency was calculated, per the manufacturer’s instructions to obtain the percent viability and IC_50_.

### OVCAR3 xenograft model

The anti-tumor activity of the anti-STn ADCs were evaluated as single agents in an OVCAR3 ovarian cancer cell line derived xenograft model. This protocol was reviewed and approved by Translational Drug Development (TD2) Institutional Animal Care and Use Committee (IACUC) and in accordance with the National Institutes of Health Guide for the Care and Use of Laboratory Animals (NIH Publications No. 80–23, revised 1996). Humane endpoints were used in addition to study endpoints. Conditions such as loss of body weight > 20% for greater than 1 week, tumors that prohibit normal physiologic function or bleed or produce exudate, prolonged diarrhea or respiratory distress were all endpoints that were assessed for daily and mice were weighed twice weekly. No analgesics were administered to tumor bearing animals and no mortality occurred outside of planned euthanasia or humane endpoints. Mice were sacrificed at study endpoints via isoflurane inhalation.

Five million tumor cells were injected into the subcutaneous right flank (1:1 Matrigel:media mixture) into 5–8 week old female athymic nude mice. Tumors were grown to a mean tumor size of 175–225 mm^3^, or ~500 mm^3^ for large tumor treatment studies. Mice were randomly equilibrated by tumor size into treatment groups. The ADCs were diluted to appropriate concentrations using vehicle control buffer (PBS pH 7.4). Mice were injected IV with 1, 2.5 or 5 mg/kg therapeutic antibody, 5 mg/kg isotype-MMAE control, or vehicle only as single agents once weekly for five weeks (QWx5). Tumor volume and body weight were calculated twice weekly and the study was concluded when average tumor volume in the vehicle-treated group was ~1400 mm^3^ (IACUC endpoint 3000 mm^3^). One way Anova and Tukey’s posthoc analysis or student’s *t*-test were used to establish significance. P values that were less than 0.05 were considered significant.

### Ovarian patient derived xenograft model

A patient derived xenograft (PDX) model was evaluated for ADC efficacy (The START Center for Cancer Care (San Antonio, TX)). A tumor fragment (~70 mg) was subcutaneously implanted into 6–12 week old female nude mice. Tumors were grown to a mean tumor size of 200–250 mm^3^ and mice were randomly equilibrated by tumor size into treatment groups. The ADCs were diluted to appropriate concentrations using vehicle control buffer (PBS pH 7.4). Mice were injected IV with 5 mg/kg therapeutic antibody, 5 mg/kg isotype-MMAE control, or vehicle only as single agents once weekly (QWx4). A group of mice received cisplatin IP at 3 mg/kg to compare standard of care therapy. Tumor volume and body weight were assessed twice weekly and mice were terminated when average tumor volume for the group exceeded 1000 mm^3^ or at the end of the study (IACUC endpoint 2500 mm^3^). No mice were sacrificed humanely prior to this endpoint. One way Anova and Tukey’s posthoc analysis was used to establish significance. P values that were less than 0.05 were considered significant.

### Immunohistochemistry for STn expression

For assessment of STn expression in patient-derived tumor samples, a selection of FFPE PDX samples (The START Center for Cancer Care) were stained with anti-STn or isotype control mAbs to determine reactivity with neoplastic cells. PDXs that were utilized had been declassified by the manufacture, and patient donors were not identifiable. Optimal IHC staining conditions were determined and validated on appropriate STn-positive and STn-negative control cells and tissues prior to testing. The IHC-stained PDX samples were evaluated and scored for cell type, subcellular localization, and staining intensity and frequency. Samples stained positively for STn were chosen for subsequent *in vivo* xenograft assessment studies. For assessment of STn expression in xenograft models post treatment, tumors were excised and paraffin embedded prior to staining with anti-STn mAb or isotype control.

### Tissue cross-reactivity

This study examined binding of the anti-STn mAb 2G12-2B2-L0H3 to a panel of normal human and cynomolgus monkey tissues and was performed at Alizée Pathology (Thurmont, MD). A human IgG1 isotype control and assay control (no primary antibody) were included in the study. A series of staining runs were performed utilizing a range of conditions and antibody concentrations to determine the optimal antibody concentrations for maximal positive control staining and minimal background staining. Method development staining runs were performed using an indirect immunoperoxidase procedure. To prevent non-specific reactivity between the secondary antibody (biotinylated donkey anti-human IgG) and IgG1 endogenous to the tissues being examined, method development was performed by pre-complexing the primary and secondary antibodies overnight prior to staining. The study was conducted on frozen sections at the optimal concentration of 3 ug/ml.

### Cynomolgus monkey toxicology evaluation

All toxicology studies were performed at Charles River Laboratories (Shrewsbury, MA) and were approved by the Institutional Animal Care and Use Committee (IACUC). Studies complied with all applicable sections of the Final Rules of the Animal Welfare Act regulations (Code of Federal Regulations, Title 9), the Public Health Service Policy on Humane Care and Use of Laboratory Animals from the Office of Laboratory Animal Welfare, and the Guide for the Care and Use of Laboratory Animals from the Institute for Laboratory Animal Research, National Research Council. This site has an approved Assurance Statement (D16-00496 [A3863-01]) on file with the Office of Laboratory Animal Welfare (OLAW), National Institutes of Health and is accredited by the Association for Assessment and Accreditation of Laboratory Animal Care International (AAALAC International) (unit 000125). Animals were socially housed in stainless steel perforated floor cages with an automatic watering system and with primate chow provided twice daily in amounts appropriate to size and age of the animals. Animals were additionally provided environmental enrichment such as perches, floor toys, foraging devices, music, and natural sounds.

To determine the potential toxicity of 2G12-2B2-L0H3-MMAE, the test article was administered by intravenous bolus injection for a total of 2 treatments (Days 1 and 22) at 1, 3, or 6 mg/kg. At the initiation of dosing, monkeys were approximately 2 to 4 years of age and weighed between 2.1 to 2.7 kg. The following in-life and laboratory procedures were performed: mortality/moribundity checks (twice daily), clinical observations, body weights, hematology, coagulation, clinical chemistry, bioanalysis and toxicokinetic evaluation. Animals were examined by veterinary staff as warranted based on observations so that appropriate interventions could be taken to minimize suffering and distress. Humane endpoints were in place, and animals experiencing severe or chronic pain/distress that cannot be relieved would be humanely euthanized. For PK analysis and half-life determination, blood (2mL) was collected at various time points from all groups following dosing on Days 1 and 22 of each administration: 1, 3, 6, 24, 48, 72, 96, 168, 240, 336 and 504h. Whole blood was processed to serum and analyzed for IgG content using a standard anti-human IgG ELISA with WinNonlin software utilized for PK and half-life calculations. On Day 29, all animals were sedated using Ketamine HCl prior to being euthanized by intravenous injection followed by exsanguination. No animals were euthanized prior to this endpoint in this study.

## Results

### Humanization of 2G12-2B2 and 5G2-1B3

**Fig 1:**
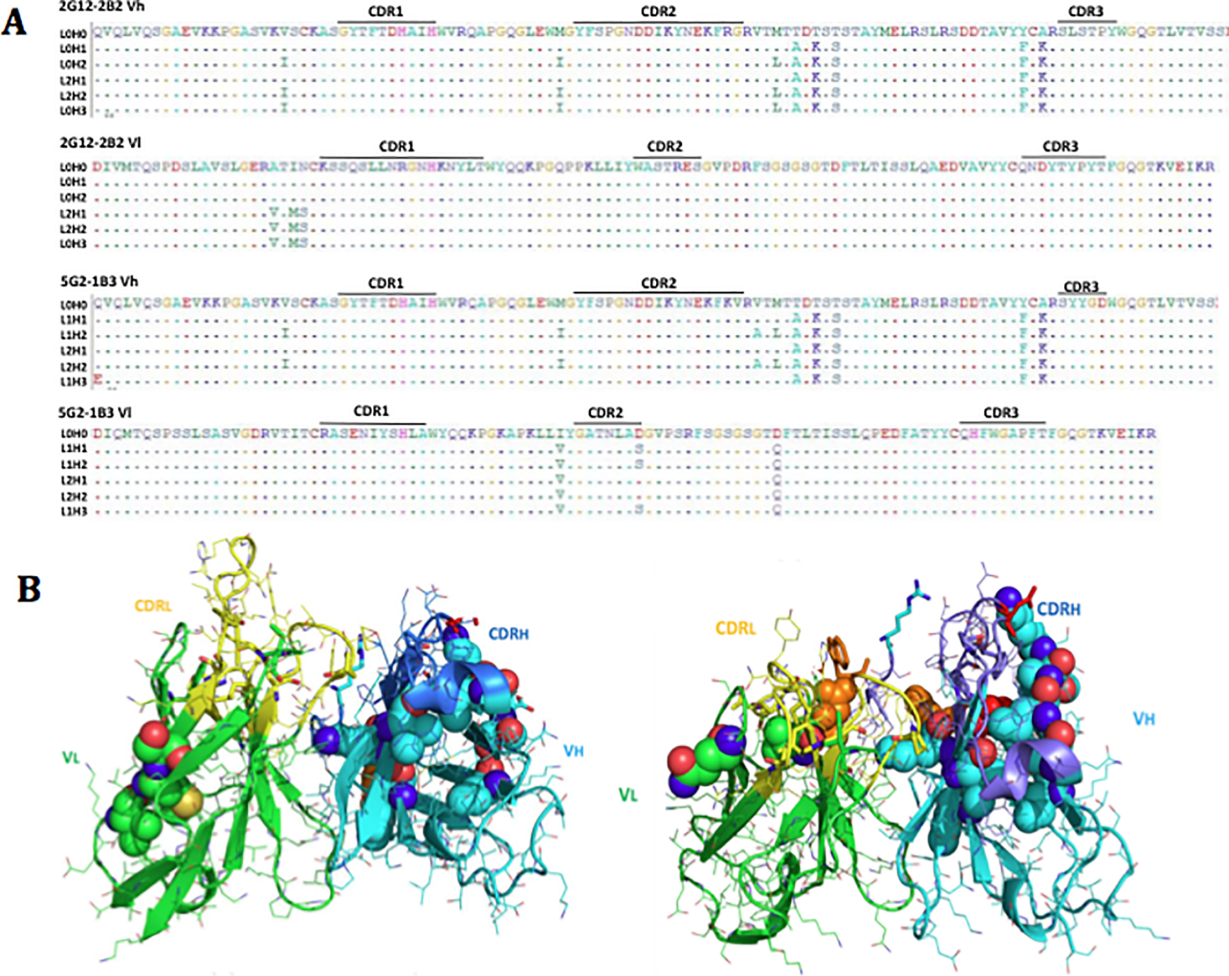
Sequence alignment and 3D structures of 2G12-2B2 and 5G2-1B3 humanized variants. (A) Sequence differences between each humanized variant and CDR grafts (L0H0) are shown. (B) 3D composite structures of humanized 2G12-2B2 (left) and 5G2-1B3 (right) variants with back-mutations visualized as spheres.

Two murine antibodies[30] were selected for humanization: 2G12-2B2 and 5G2-1B3. Mouse variable germline genes for 2G12-2B2 and 5G2-1B3 were identified as muIGHVIS53/muIGKV8-19 and muIGHV1S53/muIGKV12-46, respectively. Human acceptor frameworks were selected based on their sequence homology and robust pairing propensities: 2G12-2B2 IGHV1-18x01, IGKV4-1x01, 5G2-1B3 IGHV1-18x01, IGKV1-29x01. Multiple humanized variants were selected for production: 2G12-2B2 L0H0, L0H1, L0H2, L2H1, L2H2 and L0H3; 5G2-1B3 L0H0, L1H1, L1H2, L2H1, L2H2 and L1H3 (Fig 1A). Back-mutations in the 2G12-2B2 variants (Fig 1B left) and 5G2-1B3 variants (Fig 1B right) were introduced to preserve murine amino acids necessary to retain paratope conformation, to reduce manufacturing liabilities and to maintain antibody stability[31]. The complete panel of back-mutations is shown in S1 Table.

### Characterization of humanized mAbs

**Fig 2:**
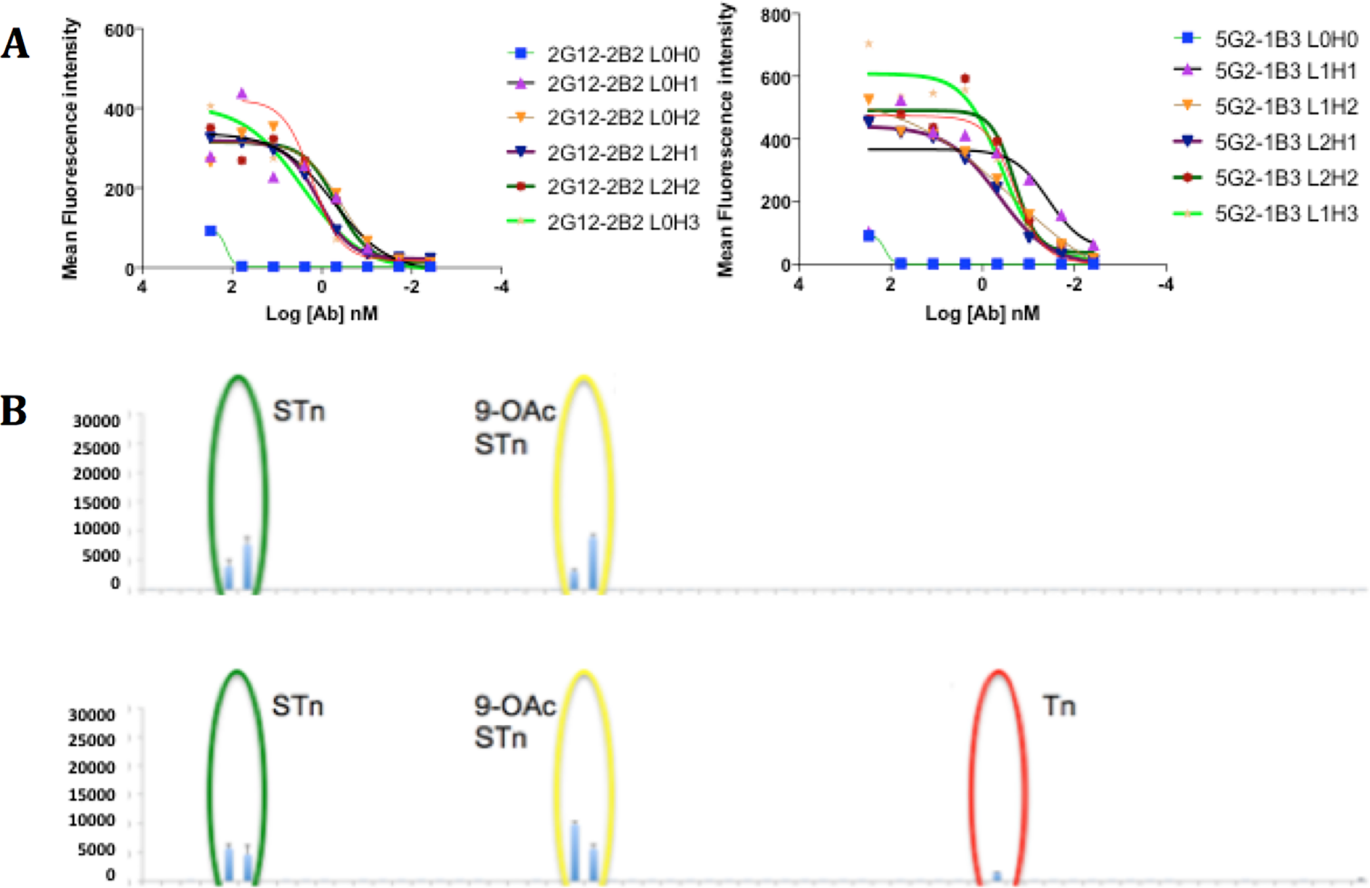
Binding affinity, cytotoxicity and selectivity of humanized mAbs. (A) 2G12-2B2 L0H3 (left) and 5G2-1B3 L1H2 (right) flow cytometric results show binding to STn expressing MDA-MB-231 STn+ cells. (B) Humanized mAbs 2G12-2B2 L0H3 (top) and 5G2-1B3 L1H2 (bottom) binding selectivity for STn and Tn by glycan array.

To characterize the antibodies after humanization, a panel of humanized 2G12-2B2 and 5G2-1B3 variants were tested by flow cytometric binding to STn-expressing MDA-MB-231 cells, as was performed previously with the murine parental antibodies[30]. Antibody binding affinity (<10 nM) was retained across all humanized constructs except for L0H0, the CDR graft control of each antibody (Fig 2A). There are two distinct forms of sialic acid, Neu5Ac and Neu5Gc, both of which may serve as tumor targets[32,33] and which may be 9-O acetylated. Assessment of glycan selectivity with our glycan array demonstrated specific binding of 2G12-2B2 variants to all four variants of STn (Neu5Ac, Neu5Gc and the corresponding 9-O acetylated forms) (Fig 2B top). Additionally, 5G2-1B3 variants demonstrated low level binding to non-sialylated Tn, another reported tumor glycomarker (Fig 2B bottom). With binding affinity and a more selective profile, 2G12-2B2 L0H3 was chosen to be carried forward for further development to minimize murine back-mutations in the humanized framework while retaining mutations that removed potential manufacturing liabilities.

### Humanized anti-STn ADCs are selectively cytotoxic to tumor cells expressing STn

**Fig 3:**
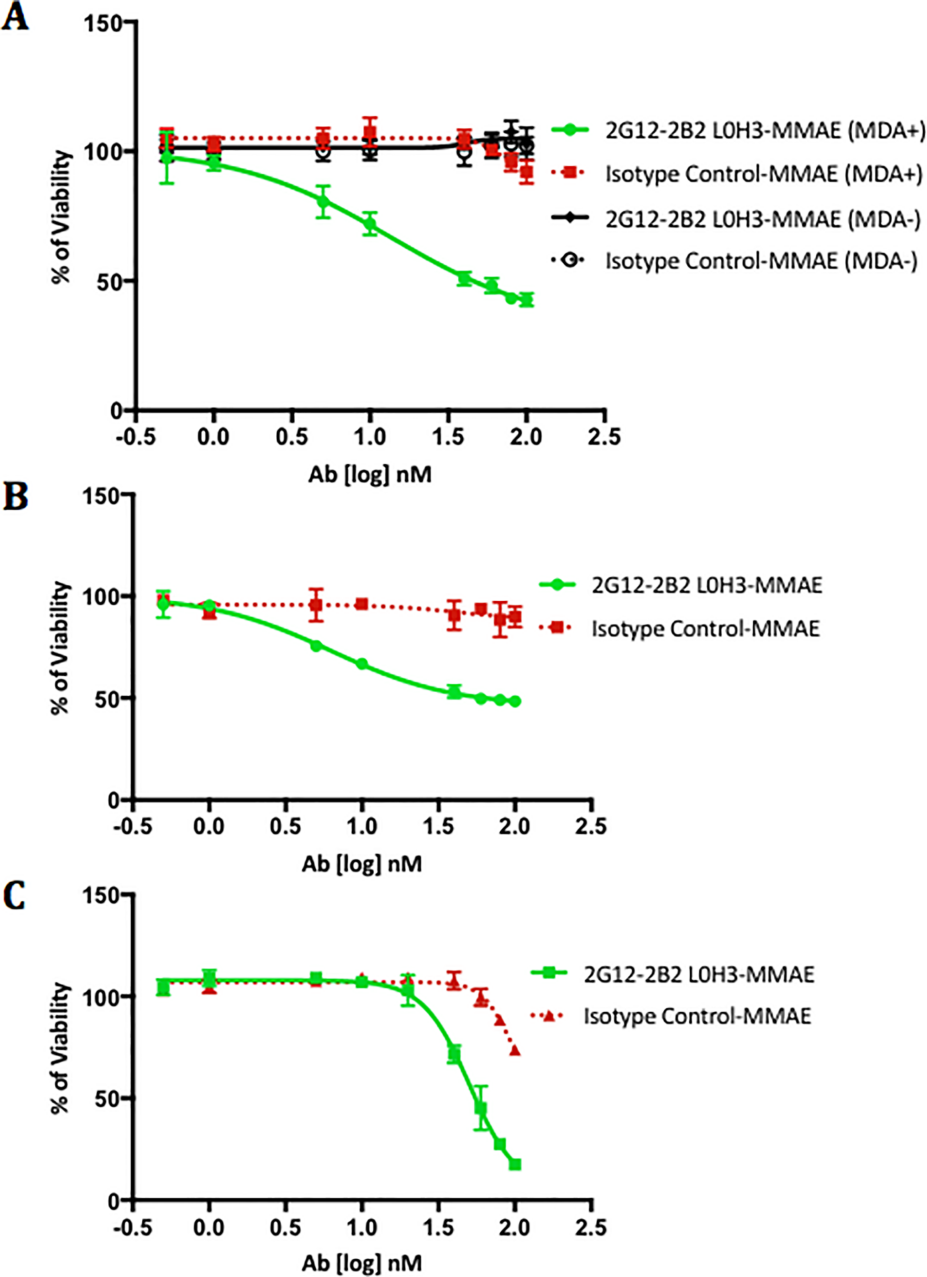
Humanized anti-STn ADCs inhibit *in vitro* carcinoma growth. Parental and engineered MDA-MB-231^STn+^ breast cancer cells (A), OV90 ovarian carcinoma cells (B) and OVCAR3 ovarian carcinoma cells (C) were treated with anti-STn or isotype-control antibody drug conjugates over 72 hours to evaluate inhibition of proliferation as a function of STn expression.

We have previously demonstrated that conjugation to a toxin confers target-specific *in vitro* cytotoxicity to a murine anti-STn antibody[30], and have confirmed those findings utilizing the ovarian cancer cell line, SKOV3, engineered to overexpress STn. To demonstrate that our humanized mAbs specifically kill cells expressing STn, we utilized the breast cancer cell line, MDA-MB-231, that has been shown to be STn negative as well as an engineered MDA-MB-231 cell line that expresses STn[23]. Treatment of MDA-MB-231 cells with the 2G12-2B2 L0H3-MMAE ADC for 72 hours induced significant cell death in cells that express STn (IC_50_ 13.6 nM) compared to those without STn (IC_50_ is non-quantifiable) demonstrating that cytotoxicity is STn target specific (Fig 3A). We additionally tested two established ovarian carcinoma cell lines OV90 (Fig 3B) and OVCAR3 (Fig 3C) which display high and moderate STn cell surface expression respectively[30]. OV90 IC_50_ 6.3 nM and OVCAR3 IC_50_ 50.5 nM suggest that cytotoxicity of the ADC is dependent upon the extent of STn expression on the target cell and that ovarian carcinoma is a suitable tumor type for treatment with this ADC.

### Anti-STn ADC efficacy *in vivo* in ovarian cell line- and patient-derived xenograft models

**Fig 4:**
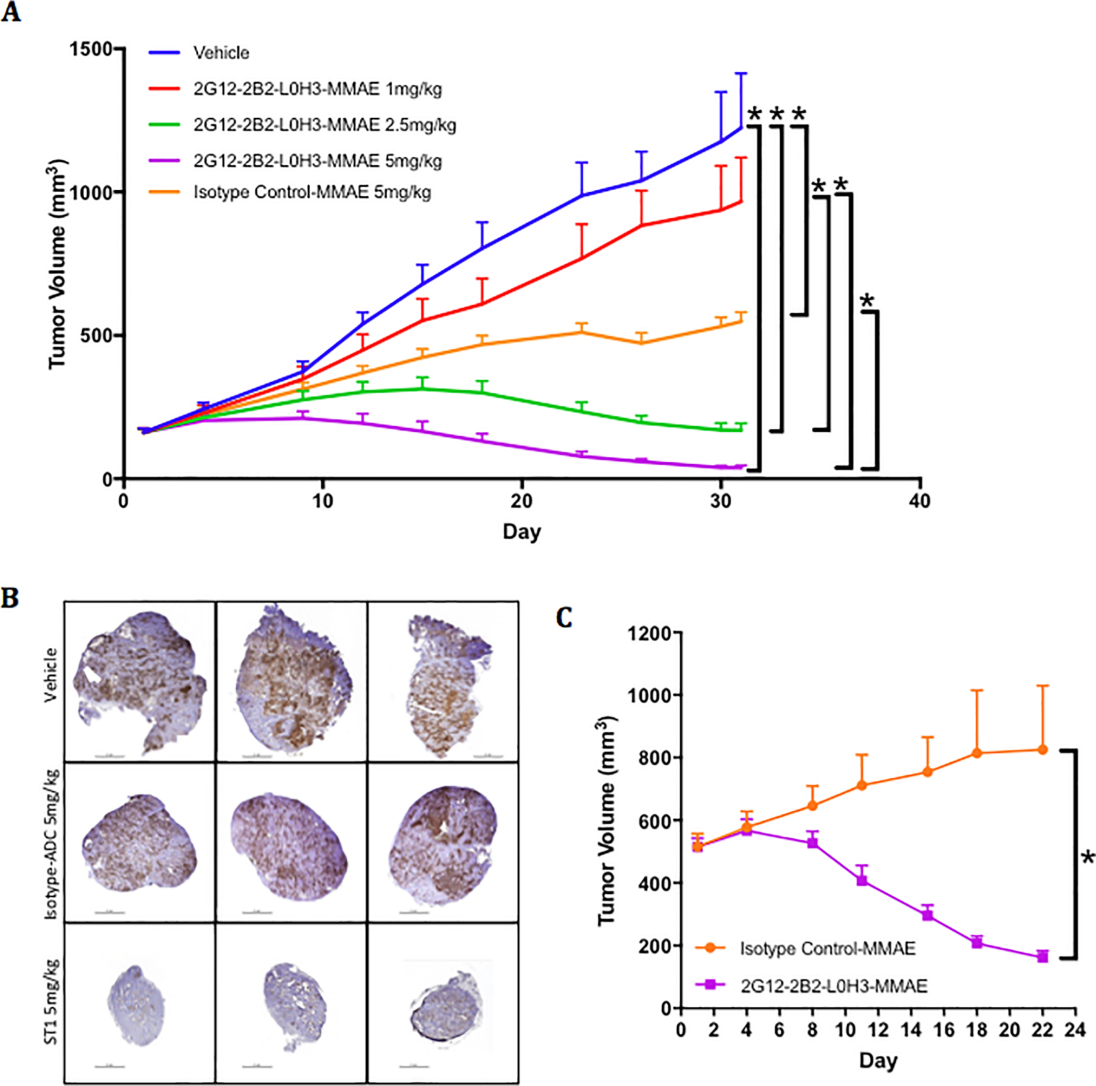
Humanized anti-STn ADCs inhibit tumor growth in OVCAR3 xenograft models. (A) Weekly dosing of OVCAR3 xenografts was initiated at Day 0 with 1, 2.5 or 5 mg/kg 2G12-2B2-L0H3-MMAE and 5 mg/kg Isotype Control-MMAE. Groups were terminated when average size of the vehicle-treated group reached ~1400 mm^3^. * P < 0.05. (B) IHC evaluation of STn expression in vehicle, isotype control-MMAE (5 mg/kg) and 2G12-2B2-L0H3-MMAE (5 mg/kg) treated groups after study termination, (C) Evaluation of treatment of larger tumors (500 mm^3^) by 5 mg/kg isotype control-MMAE and 2G12-2B2-L0H3-MMAE.

We first performed single-dose mouse PK studies of 2G12-2B2 LOH3-MMAE and determined a T_1/2_ of 2.55 days. This terminal elimination half-life data is in-line with similar published data (anti-5T4 ADC, 3.5 days)[34] and supports weekly dosing. To determine whether the demonstrated decrease in proliferation *in vitro* in OVCAR3 could be translated to a reduction in tumor size *in vivo*, a cohort of athymic nude mice harboring OVCAR3 cell line-derived xenograft tumors were treated with 2G12-2B2 L0H3-MMAE and evaluated as single agents. Mice treated with 2G12-2B2 L0H3-MMAE at 1, 2.5 and 5 mg/kg showed a percent tumor growth inhibition (TGI) (%T/C) of 79%, 13.8% and 3.2%, respectively. All groups except for 1 mg/kg were found to be significant compared to vehicle to P<0.01 (Fig 4A). Treatment at 5 mg/kg was found to be significantly (P<0.01) more potent than a corresponding isotype control-MMAE dose, demonstrating that the therapeutic effect of anti-STn ADCs is target-specific. Immunohistochemistry (IHC) staining for STn on paraffin embedded tumors at the termination of this study demonstrated loss of target expressing cells after treatment (Fig 4B). A cohort of mice whose tumors were allowed to grow to 500 mm^3^ before treatment with 5 mg/kg 2G12-2B2-L0H3-MMAE or isotype control-MMAE demonstrated that anti-STn therapy may be effective against larger, more established tumors (Fig 4C). No significant weight loss was observed for any treatment groups, indicating the therapy was well tolerated by all the groups.

**Fig 5:**
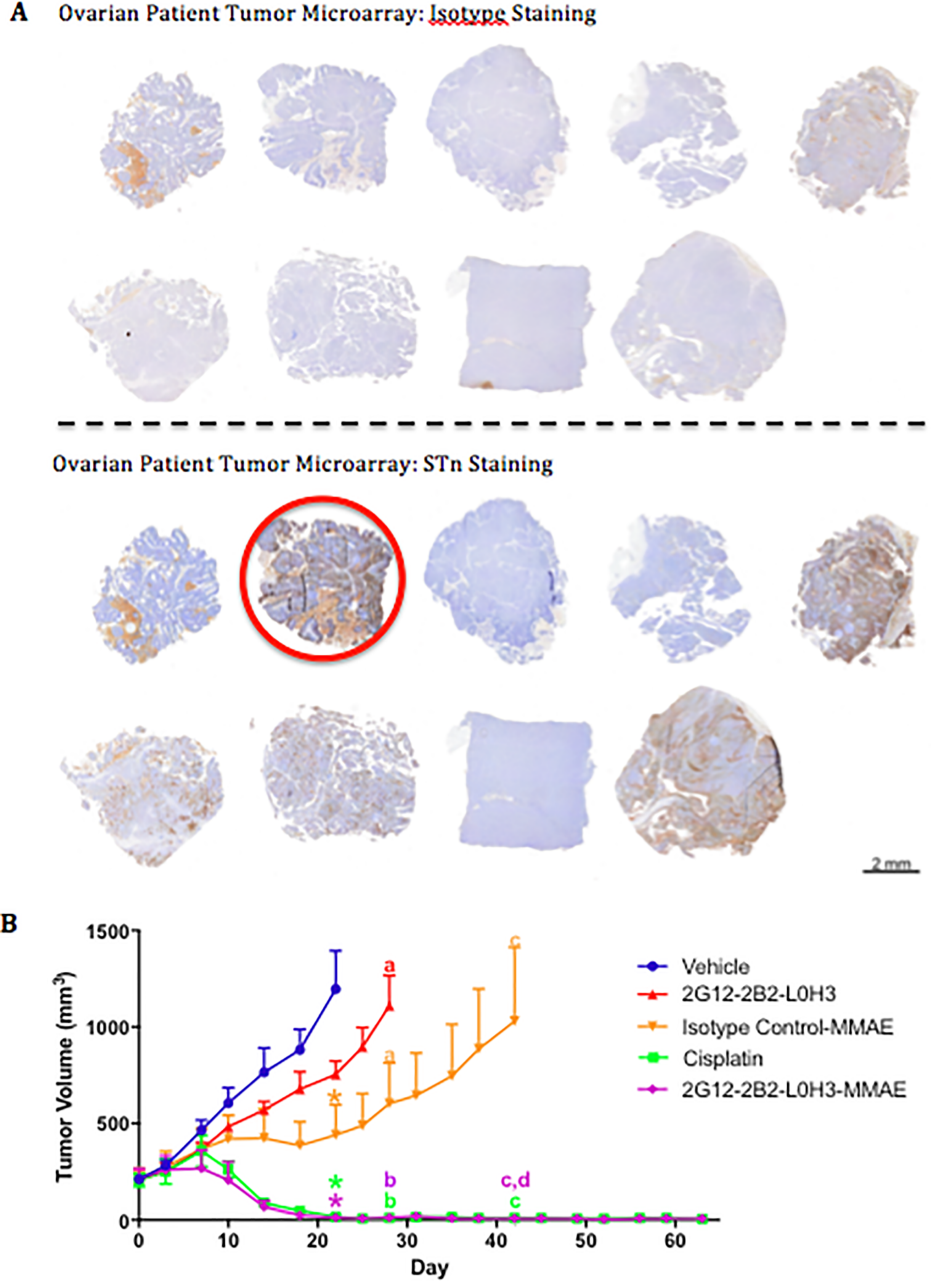
Humanized anti-STn ADCs inhibit tumor growth in a patient-derived ovarian carcinoma xenograft model. A) IHC staining for STn expression in a panel of patient ovarian tumors. The differential staining between isotype control (top) and anti-STn antibody (bottom) resulted in the selection of the patient tumor circled in red for evaluation as a PDX in an efficacy study. B) Evaluation of treatment of selected PDX model after implantation into SCID mice. ADCs were administered weekly at 5 mg/kg and cisplatin was administered at 3 mg/kg for seven weeks. Groups were terminated when average size reached 1000 mm^3^ or at the end of the study. For assessing significance, groups were compared at the termination of each arm of the study. Treatment points that do not share a symbol or a letter are significantly different: P < 0.05.

We stained a large panel of ovarian PDX tumor samples to identify STn-expressing models. Interestingly, of the serous carcinoma/adenocarcinoma samples (papillary and non-papillary) that were evaluated, 33 out of 36 samples had some level of STn positivity. One of these ovarian PDX models shown to express STn by immunohistochemistry (Fig 5A) was used for *in vivo* efficacy studies. On Day 0, mice were stratified by mean animal tumor volume and treatment was initiated QW with vehicle, 2G12-2B2 L0H3-MMAE, an isotype control-MMAE, unconjugated 2G12-2B2 L0H3, or cisplatin. Treatment with either 2G12-2B2 L0H3-MMAE or cisplatin resulted in complete tumor regression over 60 days (Fig 5B). It is important to note that with vc-MMAE ADCs, non-binding isotype controls have been reported to show variable efficacy through non-target associated toxin effects[35–37]. This effect contributes to the maximum tolerated dose observed in the clinic, and the observed increased inhibition by 2G12-2B2 L0H3-MMAE demonstrates specificity of targeting.

### Tissue cross reactivity

Human and cynomolgus monkey tissues of the heart, stomach, small intestine, colon, pancreas, and spleen were stained for STn using 2G12-2B2-L0H3 (Table 1). The study was conducted on frozen sections at the optimized concentration of 3 ug/ml (see methods). Cytoplasmic staining was noted for most organs in humans and cynomolgus monkeys, however no membrane staining was visualized in any normal tissues tested supporting that all membrane staining is tumor specific.

**Table 1:** Tissue cross reactivity of 2G12-2B2-L0H3-MMAE.

Tissue	Human	Cynomolgus Monkey
**Heart**		
Vessel	2+rare (cytoplasm)	Neg
**Stomach**		
Epithelial Cells	2–3+ freq (cytoplasm)	2–3+ occ (cytoplasm)
Vessel	Neg	2+ occ (cytoplasm)
**Lung**		
Epithelium	Neg	2–3+ freq (cytoplasm)
**Small Intestine**		
Epithelial Cells	2–4+ freq (cytoplasm)	3–4+ freq (cytoplasm)
**Colon**		
Epithelial Cells	1+ freq (cytoplasm)	Neg
Vessel	Neg	4+ freq (cytoplasm)
**Pancreas**		
Vessel	Neg	2–3+ freq (cytoplasm)
**Spleen**		
Vessel	2–3+ freq (cytoplasm)	Neg

### Cynomolgus monkey toxicity and pharmacokinetics

To evaluate toxicity in cynomolgus monkeys, 2G12-2B2-L0H3-MMAE was administered via intravenous (slow bolus) injection using syringe/needle via a peripheral vein on Days 1 and 22 at 1, 3, and 6 mg/kg. All animals survived until scheduled termination. There were no 2G12-2B2-L0H3-MMAE related clinical observations noted or variations in the body temperature, bodyweight, and coagulation and clinical chemistry parameters. Changes in hematology parameters consisted of minimally to mildly decreased red blood cell mass (hemoglobin, red blood cell count, and hematocrit) and red cell distribution width at 6 mg/kg with associated decreased or inadequately increased reticulocytes at ≥ 3 mg/kg. Changes in leukocyte parameters consisted of mildly to moderately decreased neutrophils, monocytes, and total white blood cell count followed by mild to moderate increases for these leukocytes at 6 mg/kg with subsequent recovery, and transient decreased large unstained cells at 6 mg/kg. Histopathologic changes were limited to the bone marrow. 2G12-2B2-L0H3-MMAE related changes in bone marrow cytology included decreased myeloid:erythroid ratio at 6 mg/kg (increased proportion of erythroid lineage cells relative to myeloid lineage cells). Based on these results, the no-observed-adverse-effect level (NOAEL) was considered to be 1 mg/kg (Day 22: mean AUC(0-t) at 986 hr*ug/mL and Cmax at 22.2 ug/mL). See Table 2 for full pharmacokinetic characterization.

**Table 2:** Cynomolgus monkey pharmacokinetic parameters.

Mean PK parameters post the first dose														
Dose (mg/kg)	C_max_ (ug/mL)		AUC_last_ (Day*ug/mL)		AUC_0-7 Day_ (Day*ug/mL)		AUC_INF_ (Day*ug/mL)		CL (mL/kg/day)		HL (Day)		V_SS_ (mL/kg)	
	Mean	SD	Mean	SD	Mean	SD	Mean	SD	Mean	SD	Mean	SD	Mean	SD
1	19.13	1.78	57.31	7.5	42.6	0.96	62.62	7.81	16.13	1.96	4.66	1.43	96.83	17.29
3	81.24	11.17	228.39	25.02	157.34	4.94	267.39	56.39	11.57	2.52	7.47	3.91	96.16	30.64
6	151.37	20.97	419.05	50.18	342.22	32.58	440.63	28.26	13.65	0.86	3.53	0.51	61.62	10.98

## Discussion

Ovarian cancer is the leading cause of death from gynecological malignancies in the U.S. It is estimated that 22,440 women will be diagnosed with ovarian cancer and 14,080 will succumb to this disease in 2017, making it the fifth leading cause of cancer deaths in women[1]. This high mortality can be ascribed to non-symptomatic onset, late-stage diagnosis, cancer aggressiveness, a high degree of tumor heterogeneity and a general lack of therapeutically targetable genetic changes[38,39]. The current standard of care is surgical debulking followed by taxane- and platinum-based chemotherapy. While this initial treatment results in ~70% of patients achieving an initial complete clinical response, the majority of these patients will relapse with chemoresistant disease[1]. Ovarian cancer has few common targetable mutations, amplifications and/or deletions. *TP53* is the most commonly mutated gene in ovarian cancer[2], however, strategies for targeting p53 have been unsuccessful. The next most commonly altered pathway that has been identified is the Notch pathway[40], though targeting members of the Notch pathway have only had limited success[41]. The percentage of tumors impacted by any other particular genetic alteration besides p53 and the Notch pathway is minimal[2]. Therefore, the identification of additional alterations in ovarian cancer is key to developing effective, targeted therapies.

Glycosylation of proteins is one of the most abundant and diverse post-translational modifications, with more than half of all human proteins estimated to be glycosylated[42]. Interestingly, aberrant glycosylation is present in tumors more frequently than oncogene products and the association of tumor glycosylation with tumor progression may be stronger than the deletion or inactivation of tumor-suppressing genes[43]. Therefore, targeting an altered glycosylation pattern specific to tumor cells may offer significant anticancer benefit.

STn is found on a significant number of ovarian cancers and is expressed on the well-known ovarian cancer biomarker CA-125 (MUC16), as well as on MUC1[44,45], and is rarely present on normal tissue. Furthermore, levels of these STn-associated mucins in serum have been used to differentiate cancerous versus benign ovarian disease[46]. Elevated serum levels of STn occur in about 50% of ovarian cancer patients and correlate with lower progression-free survival and overall five-year survival rates[47]. Furthermore, Chen *et al* have reported that the use of STn MUC16 detection instead of the general CA-125 assay led to an increase in assay specificity[48]. The potential importance of STn in cancer has been discussed previously and there have been attempts to target it with a cancer vaccine. Theratope (Biomira, Inc. (Edmonton, Alberta, Canada)) was a multi-valent STn-based vaccine aimed at mounting a host immune response against STn-expressing cells. Although it reached phase III clinical trials for breast cancer, it did not achieve the needed efficacy to receive FDA approval[49]. Despite its initial failure, retrospective analysis suggested that there were subsets of patients that benefitted from treatment[29]. STn expression is variable in breast cancer[18], and therefore efficacy may have been improved if patients had been pre-selected for STn positivity.

Humanized anti-STn antibodies have been explored as therapeutic agents[50–52], however this report is the first study to evaluate a direct toxin conjugate to a humanized anti-STn antibody for targeting STn expressing tumors. In addition, anti-STn therapies may be particularly well suited for the treatment of a significant subset of ovarian carcinomas. The use of ADCs in treating solid tumors including ovarian cancer has been the focus of many recent investigational efforts. For example, three ADCs are currently in clinical development. Anetumab ravatansine, which targets mesothelin-positive solid tumors, is in phase II and mirvetuximab soravtansine, which targets folate receptor alpha, is in phase III development[53]. A phase I study investigating DMUC5754A which targets MUC16 with an ADC was recently completed[8]. As with any ADC therapy, screening the patient population for the presence of the targeted marker is needed in order to determine the efficacy of the drug in reducing tumor size[53]. STn is found on a number of proteins expressed on ovarian cancer cell surface including proteins such as MUC1 and CD44[18,54]. Approximately 85% of ovarian cancers express STn[18], which makes STn a favorable target for therapeutic intervention in ovarian cancer. We undertook the humanization of two murine anti-STn mAbs 2G12-2B2 and 5G2-1B3 and evaluated their *in vitro* and *in vivo* anti-tumor efficacy. The humanization process did not alter the specificity or affinity of the antibodies to STn, and as ADCs they were cytotoxic to STn-expressing ovarian cancer cells lines that endogenously express varying levels of the STn carbohydrate on their cell surfaces. *In vivo*, humanized anti-STn ADCs inhibited the growth of cancer cell line and patient derived ovarian xenograft models in mice. Further, toxicity studies in cynomolgus monkeys demonstrated only mild MMAE class-related hematological effects, and micropathology review on all major organs showed no observations linked to STn target associated toxicity.

Collectively, our data supports the potential for an STn-targeted therapy in treating ovarian cancer, a malignancy with limited currently effective therapies and unimproved survival rates over the past 20 years[1]. The known specificity of STn for malignant tissue in combination with the high affinity and STn-specific selectivity of the mAbs presented herein warrant further investigation for anti-STn ADCs as single agent or combination therapy in the clinical setting, specifically in patients with STn-expressing tumor cells.

## Supporting information

S1 Table Humanized variant mutations. (DOCX)
