# Supplementary material for: Humanized anti-Sialyl-Tn antibodies for the treatment of ovarian carcinoma
Source: PLoS One. 2018 Jul 27;13(7):e0201314. doi: 10.1371/journal.pone.0201314 (PMC6063429; doi:10.1371/journal.pone.0201314)
Supplement: S1 Table — (DOCX) [file pone.0201314.s001.docx]

Supplementary Table 1: Humanized Variant Mutations

| **Light (L) / Heavy (H)**  **Chain Variant** | **2G12-2B2 Mutations** | **5G2-1B3 Mutations** |
| --- | --- | --- |
| L1 | None | I48V, S56D, D70Q |
| L2 | A19V, I21M, N22S | I48V, D70Q |
| H1 | T71A, A93K, T73K, T75S, Y91F | A24D, T71A, T73K, T75S, Y91F, A93K |
| H2 | T71A, A93K, T73K, T75S, Y91F, M48I, M69I | A24D, T71A, T73K, T75S, Y91F, A93K, V20I, V67A, M69L, M48I |
| H3 | T71A, A93K, T73K, T75S, Y91F, Q1E | A24D, T71A, T73K, T75S, Y91F, A93K, Q1E |
